# Unexpected Serotonin Syndrome, Epileptic Seizures, and Cerebral Edema Following 2,5‐dimethoxy‐4‐bromophenethylamine Ingestion

**DOI:** 10.1111/1556-4029.14214

**Published:** 2019-10-23

**Authors:** Antoinette S. Spoelder, Jan K. G. Louwerens, Stefanie D. Krens, Nynke Jager, Natalie E. LeCouffe, Wouter de Ruijter, Tibor M. Brunt

**Affiliations:** ^1^ Department Intensive Care Northwest Clinics (Noordwest Ziekenhuisgroep) Postbus 501 1800 AM Alkmaar The Netherlands; ^2^ Amsterdam University Medical Centre Meibergdreef 9 1105 AZ Amsterdam The Netherlands; ^3^ Radboud Universiteit, Behavourial Science Institute Montessorilaan 3 6525 HR Nijmegen The Netherlands

**Keywords:** forensic science, 2C‐B, intoxication, serotonin syndrome, designer drugs, phenethylamine

## Abstract

4‐bromo‐2,5‐dimethoxyphenethylamine (2C‐B) is a designer drug. In Europe, 2C‐B is easily obtained and used for recreational purposes. It is known for its stimulating effects similar to those of 3,4‐methylenedioxymethamphetamine, although in higher doses it has more hallucinogenic effects. Here, we report a case of 2C‐B ingestion, confirmed by liquid chromatography‐tandem mass spectrometry, in an 18‐year‐old man. The neurological consequences were severe, including the development of serotonin syndrome and severe brain edema. Supportive therapy resulted in a stable condition, although, after several months, the patient still suffered from severe neurological impairment due to the drug‐induced toxicity. This case showed that 2C‐B could not be identified with the drugs of abuse screening routinely used in Dutch hospitals. The use of 2C‐B carries many risks, with potentially profound neurological damage, that both consumers and healthcare physicians are unaware of.

The designer drug 2C‐B (2,5‐dimethoxy‐4‐bromophenethylamine) is a psychoactive substance, which was first synthesized in 1974. Currently, it is used for recreational purposes. It is widely available in Europe and has appeared increasingly on the illicit drugs market over the past years 1. In the Netherlands, 2C‐B is one of the most frequently used designer drugs. In low doses, it produces enhanced sensory sensitivity and has stimulating effects similar to those of 3,4‐methylenedioxymethamphetamine (MDMA) or “ecstasy.” In higher doses, the psychedelic and hallucinogenic effects predominate. 2C‐B use is popular because of this transient effect and its association with only few intoxications or severe side effects 2, 3. We report the case of a young man who developed serotonin syndrome and severe brain edema after taking 2C‐B.

## Case Description

In the early morning, an 18‐year‐old Caucasian male with impaired consciousness was presented to the emergency department (ED) by paramedics. One hour prior to admission, the patient had been found at home by his friends, unresponsive. The patient had no previous medical history. Hetero‐anamnesis revealed that the patient used cannabis, lysergic acid diethylamide (LSD), and alcohol on a recreational basis. His friends reported there was a possibility he ingested a drug that night for recreational purposes.

Prior to admission to the ED, the patient was administered benzodiazepines by the paramedics of the emergency services. Examination of the patient revealed an obstructive breathing pattern and trismus. Vital parameters showed an oxygen saturation of 95% with 15 L O_2_, a respiratory rate of 40/min, tachycardia with a pulse of 180/min, and a blood pressure of 140/90 mmHg. The Glasgow Coma Score was 3 (E1‐M1‐V1). Pupils were slightly unequal and dilated, but responsive to light, and brain stem reflexes were present. He had no nuchal rigidity. In the ED, the patient experienced tonic seizures, with urinary incontinence and blood residue in his mouth. For this, he was given levetiracetam intravenously. The patient’s temperature was 38.5°C. The glucose level was 3.3 mmol/L. The differential diagnosis included meningitis, intoxication, epilepsy, cerebral hemorrhage, or ischemia. Because of the severe epileptic manifestations and possible neurological abnormalities, the patient was sedated and intubated.

Laboratory results showed an elevated creatine kinase (CK) of >10,000 IU/L, signs of acute kidney injury, and leucocytosis, with a normal C‐reactive protein level. Troponins were elevated (max 3.2 µg/L) without any ECG abnormalities. Liver transaminases were elevated. Arterial blood gas analysis showed a respiratory compensated metabolic acidosis. The serum anion gap was 21 mmol/L, and lactate was 4 mmol/L. The measured osmolality was 312 mmol/L; the calculated osmolality was 309 mmol/L, resulting in a normal osmol gap.

The patient’s initial cerebral computed tomography (CT) scan revealed mild cerebral edema without signs of ischemia, hemorrhage, or cerebral herniation. Because of the cerebral edema, a lumbar puncture was not attempted. As meningitis could not be ruled out, dexamethasone and intravenous antibiotics (ceftriaxone and amoxicillin) were administered. Because there were signs of rhabdomyolysis, (in the intensive care unit [ICU]) the patient underwent hyperhydration with crystalloid solution, combined with diuretics to promote urine output. Electrophoresis revealed the variant creatine kinase—MM isoenzyme subtype to be the origin of the elevated CK. This suggested that the origin of the elevated CK level was probably striated muscle tissue due to damage of the skeletal muscles 4.

One day after admission, the neurological status of the patient slightly improved (E2 M4right M3left Vtube). Cerebral magnetic resonance imaging (MRI) showed cytotoxic edema with leptomeningeal enhancement. This might indicate bacterial meningitis or nonspecific enhancement due to intoxication. The patient’s temperature rose to 39.6°C. Laboratory results showed a rising serum level of CK, peaking at 37278 IU/L. Due to recurrent seizures, intravenous anti‐epileptics were increased in dosage.

On hospital day five, the patient’s neurological status worsened, showing signs of cerebral herniation. A CT‐scan showed increased cerebral edema with obliteration of the gray/white matter interface, midline shift to the right, and narrowing of the lateral ventricles. Osmotherapy was initiated after which an emergency bilateral decompression craniectomy was performed. After surgery, the neurological status of the patient returned to E2 M4right M3left Vtube. Although the cause was likely to be a toxin (drug), based on the diagnostics at this time an infectious cause could not be ruled out. The patient was maintained on antibiotic treatment for bacterial meningitis for 14 days. In the following days, mannitol was gradually tapered to zero. The seizures remained clinically unabated; therefore, the anti‐epileptic therapy was expanded with lacosamide.

On day eleven, the patient was planned for percutaneous tracheostomy. Following the procedure, the patient’s respiratory and hemodynamic status was stable. Unfortunately, his neurological status did not improve until he was discharged to the neurology ward on day 25. Further treatment was planned in a specialized clinic for early intensive neurological rehabilitation. At follow‐up, 12 months after discharge, the patient was in a minimally conscious state and scored 5 out of 8 on the postacute level of consciousness (PALOC) scale.

### Bioanalytical analysis

Immediately following admission to the ICU, urine and blood samples were collected. Urine was screened for drugs of abuse by immunoassay (Triage^®^ TOX DRUG Screen; Alere Inc., San Diego, CA, USA), which includes amphetamines, barbiturates, benzodiazepines, cocaine, methadone, tetrahydrocannabinol, opiates, phencyclidine, and tricyclic antidepressants. This screening confirmed the presence of benzodiazepines and tetrahydrocannabinol (THC). Midazolam, a benzodiazepine, was administered in the ambulance.

In serum, a qualitative systematic toxicological screening (STIP; Ziekenhuisapotheek Noord‐Oost Brabant, Den Bosch, The Netherlands) was performed; a high‐performance liquid chromatography with photodiode array detection (HPLC‐DAD) was employed, utilizing a library of reference spectra and retention times for more than 500 different types of drugs 5. This screening did not identify any causative substances in the patient’s serum.

Because of the patient’s hetero‐anamnesis of possible use of LSD, serum was analyzed by a specific LC‐MS/MS method, but no LSD was confirmed in the patients’ sample. On day 10, a methanolic extract of the substance obtained from the patient’s dealer, provided by the police, and the patients’ serum sample on the day of admission were analyzed with an automated forensic toxicology screening method (Ziekenhuisapotheek Onze Lieve Vrouw Gasthuis, locatie oost, Amsterdam, The Netherlands). This method combines liquid chromatography‐tandem mass spectrometry with a library of over 830 compounds based on retention time, MS, MS^2^, and MS^3^ ion trap spectra (Toxtyper™; Bruker Daltonik GmbH, Bremen, Germany). In both samples, the designer drug 2C‐B was identified.

## Discussion

This case is indicative of an adverse neurological reaction to the designer drug 2C‐B, resulting in the development of a serotonin syndrome, epileptic seizures, and severe brain edema. To our knowledge, this is the first report to show this complication in relation to 2C‐B use.

**Figure 1:**
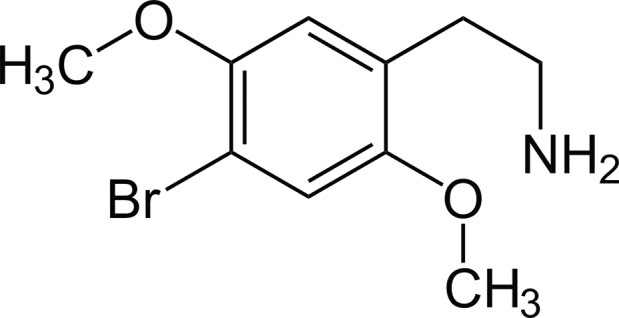
Chemical structure of 2C‐B (2,5‐dimethoxy‐4‐bromophenethylamine).

The drug 2C‐B is a phenethylamine and was first synthesized in 1974. As a class, these synthetic phenethylamines are referred to as 2C drugs. By modification of the basic phenethylamine structure, 2C‐B (Fig. 1) is synthesized 6, 7, 8. Although 2‐CB is widely used, there is only limited data about its pharmacologic profile and even less on toxicity. The 2‐dimethoxyphenethylamine part of the drug is an agonist of the serotonergic 5HT_2A_ receptor, which can cause profound hallucinations. 2C‐B also acts on several other receptors, including 5HT_2C_, 5HT_2B_, and α1‐adrenergic receptors. After oral ingestion of 10–30 mg 2C‐B, the onset of the effect is seen within 30–75 min and generally lasts 4–8 h 6, 9.

2C‐B displays a dose‐response curve with lower doses resulting in stimulating effects with increased visual, auditory and tactile sensations, whereas in higher doses, the hallucinogenic effects prevail 1, 10. 2C‐B is metabolized by liver hepatocytes, primarily by oxidative deamination followed by demethylation 11.

The ingested dose of 2C‐B was uncertain in this case, and it was not possible to quantify 2C‐B in the serum sample. Literature suggests there is a large inter‐individual difference in pharmacological response to 2C‐B, which could be due to genetic variability of enzyme‐mediated monoamine oxidase‐A and B by which deamination mainly occurs 12. Together with the drug’s pharmacological profile, this could possibly explain the severity of the toxic effects of the drug in our patient.

In this case, our patient showed neurological deterioration due to severe brain edema after the ingestion of 2C‐B. Even after several months, the patient was still suffering from severe neurological impairment due to the drug‐induced toxicity. The presence of hyperthermia and diaphoresis, tachycardia, hypertension, dilated pupils, trismus, hyperreflexia of the lower extremities and lowered Glasgow Coma Scale on admission met the criteria for serotonin syndrome using the Hunter Toxicity Criteria Decision Rules 13. To our knowledge, this is the first case report of serotonin syndrome after ingestion of 2C‐B, based on 2C‐B’s mechanism of action this can be explained by its profound action on postsynaptic 5HT_2A_ receptors.

Although there is a lack of literature on cases on serotonin syndrome after ingestion of 2C‐B, there has been a case report of a patient who developed recurrent seizures and serotonin syndrome after the ingestion of the liquid form of 2C‐I (2,5 dimethoxy‐4‐iodophenethylamine), a close analogue of 2C‐B 14. Another case described seizure after ingestion of 2C‐I, 2C‐E and 2C‐B 15. The development of brain edema is described following acute methamphetamine intoxication in rats, suggesting brain hyperthermia leads to breakdown of the brain‐blood‐barrier, which increases permeability and results in rapidly developing brain edema 16.

This case showed that 2C‐B could not be identified with the drugs of abuse screening routinely used in Dutch hospitals. Although 2C‐B’s core structure, a phenylethylamine, is shared among amphetamines, catecholamines, and cathinones, it does not cross‐react with the substrate used for the amphetamine immunoassay 17.

The purpose of this case report is to inform and add to the clinical knowledge about the effects and potential severe consequences of recreational use of 2C‐B, and the diagnostic challenges physicians encounter during the treatment of similar patients. One should be aware that common toxicological screening methods are unable to detect several new psychoactive substances (NPS), such as 2C‐B.
